# Cerebral Arteriovenous Malformation With Ipsilateral Middle Cerebral Artery Occlusion: A Case Report

**DOI:** 10.7759/cureus.51193

**Published:** 2023-12-27

**Authors:** Masahiro Yada, Ko Matsuda, Masahiko Kitano, Yoshiyasu Iwai, Shinsuke Tominaga

**Affiliations:** 1 Neurosurgery, Tominaga Hospital, Osaka, JPN

**Keywords:** cyst formation, gamma knife radiosurgery, feeder aneurysm, occlusion of the major feeding artery, cerebral arteriovenous malformation

## Abstract

We report the case of a 29-year-old man who presented with a sudden headache. Computed tomography showed a small intraventricular hemorrhage in the left lateral ventricle. Cerebral angiograms suggested rupture of a coexisting feeder aneurysm in the left temporal cerebral arteriovenous malformation (AVM). The left proximal middle cerebral artery, a major feeding artery, was occluded near the AVM, with development of abnormal blood supply, such as in moyamoya-like vessels to the nidus. After endovascular embolization of the coexisting feeder aneurysm and feeding arteries, the patient underwent volume-staged Gamma Knife radiosurgery (GKS). Follow-up angiograms performed 4.5 years after the last GKS confirmed complete disappearance of the AVM. Around 4.8 years after GKS, the patient required surgical intervention to develop delayed cyst formation; however, the postoperative course was uneventful.

## Introduction

Arteriovenous malformations (AVMs) are a vascular aggregation known as a nidus with a major arterial feeder that connects directly to the draining veins. Brain AVMs are of particular concern due to the inherent high risk of abnormal blood vessel hemorrhage, which can cause neurological impairment. There are very few reports on cerebral AVM with occlusion of the major feeding arteries, with only 15 cases of AVM with major feeding artery occlusion being reported to date [1-6]. Herein, we present a case of AVM associated with ipsilateral major artery occlusion and discuss its treatment strategy.

## Case presentation

**Figure 1:**
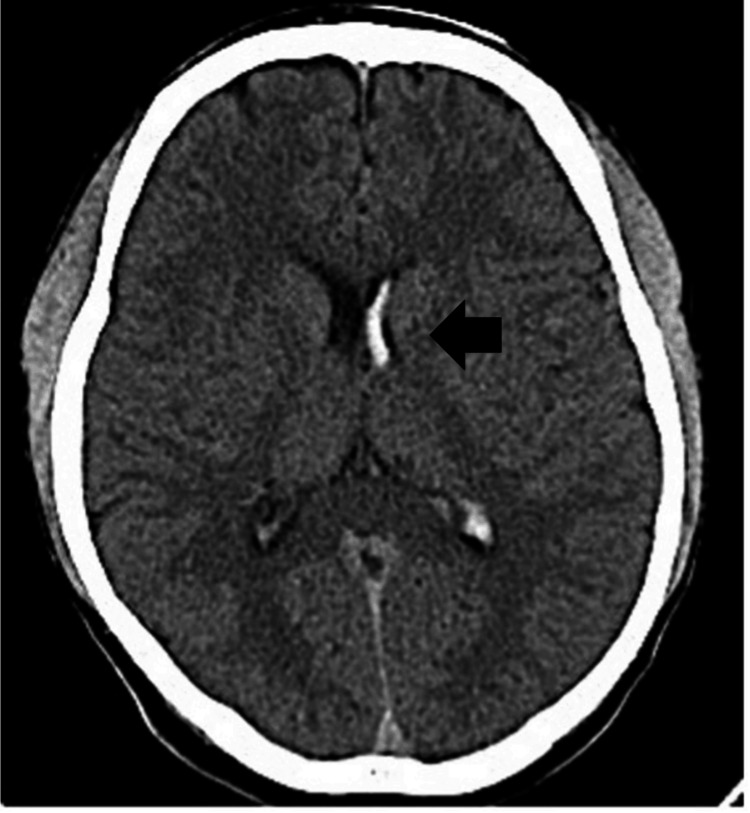
Axial image of a noncontrast computed tomography scan at the onset showing a small intraventricular hemorrhage (black arrow) at the left lateral ventricle.

A 29-year-old man presented with a complaint of a sudden headache and was transferred to a local hospital. His medical history was unremarkable except for nine years of smoking 20 cigarettes per day. Computed tomography (CT) showed a small intraventricular hemorrhage (IVH) in the left lateral ventricle (Figure 1).

**Figure 2:**
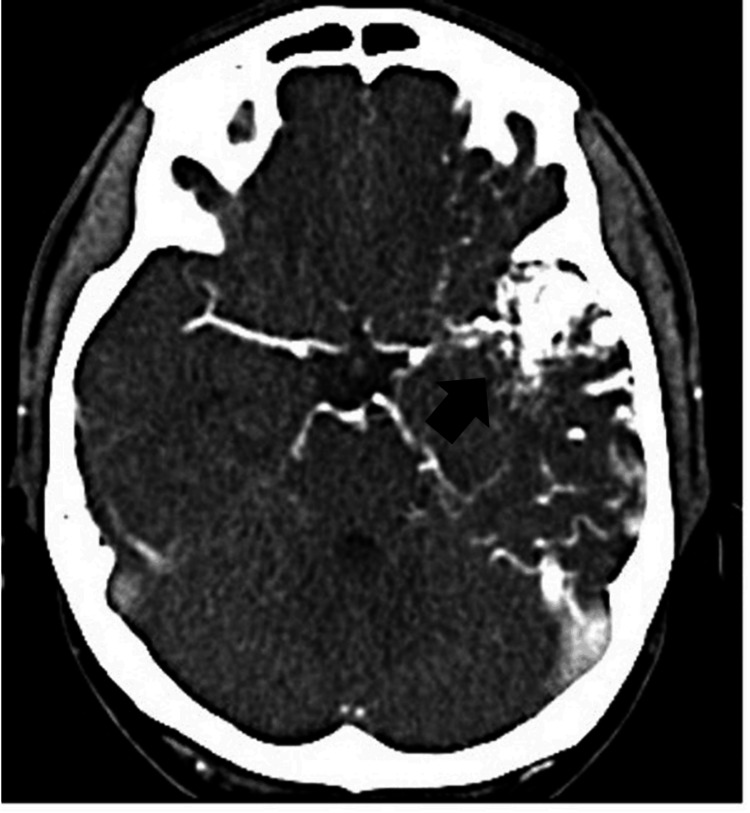
Contrast-enhanced computed tomography showing a cerebral arteriovenous malformation (black arrow) with a maximum diameter of 32 mm within the left temporal lobe.

The patient was transferred to our department for further evaluation and treatment two weeks after onset. There were no symptoms on admission, and no abnormal neurological findings were noted. Contrast-enhanced CT revealed an AVM with a maximum diameter of 32 mm in the left anterior superior and middle temporal gyri (Figure 2).

**Figure 3:**
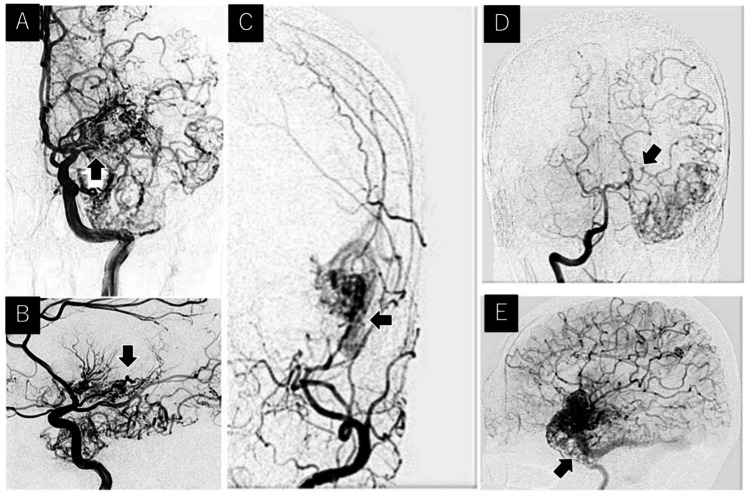
Pre-embolization DSA images of the feeders, nidus, and drainers. (A) Anteroposterior views of the ICAG showing a prominent feeder from the left anterior cerebral artery, MCA, and AchA. The proximal left MCA to the nidus exhibits occlusion with moyamoya-like vessel around it (black arrow). (B) Lateral views of the pre-embolization DSA image demonstrating a feeder aneurysm of AchA (black arrow). The proximal left MCA to the nidus exhibited occlusion with small moyamoya vessel-like arteries around it without steno-occlusive lesions around terminal portions of the internal carotid artery (ICA). (C) Anteroposterior views of the left external carotid angiography showing a prominent left middle meningeal artery feeder (black arrow). (D) Anteroposterior views of the right vertebral angiography showing a prominent left posterior cerebral artery feeder (black arrow). (E) Lateral views of the left ICAG showing venous drainage into the left transverse sinus (black arrow) through the two ipsilateral superficial cerebral veins via the sphenopetrous vein. DSA, digital subtraction angiography; ICAG, internal carotid angiography; MCA, middle cerebral artery; AchA, anterior choroidal artery

Digital subtraction angiography confirmed that the AVM was supplied by branches of the ipsilateral anterior cerebral artery, posterior cerebral artery, middle cerebral artery (MCA), anterior choroidal artery (AchA), and middle meningeal artery (MMA). The proximal MCA of the nidus showed occlusion with hyperplasia of the moyamoya-like vessels. A feeder aneurysm was found in the left AchA. The aneurysm was 1.5 mm in size and located in the inferior horn of the left ventricle. A hematoma was presented from the inferior horn of the left ventricle to the body of the left ventricle, and we considered that the ruptured aneurysm caused IVH. The draining vein returns to the transverse sinus via the ipsilateral sphenobasal vein (Figure 3).

**Figure 4:**
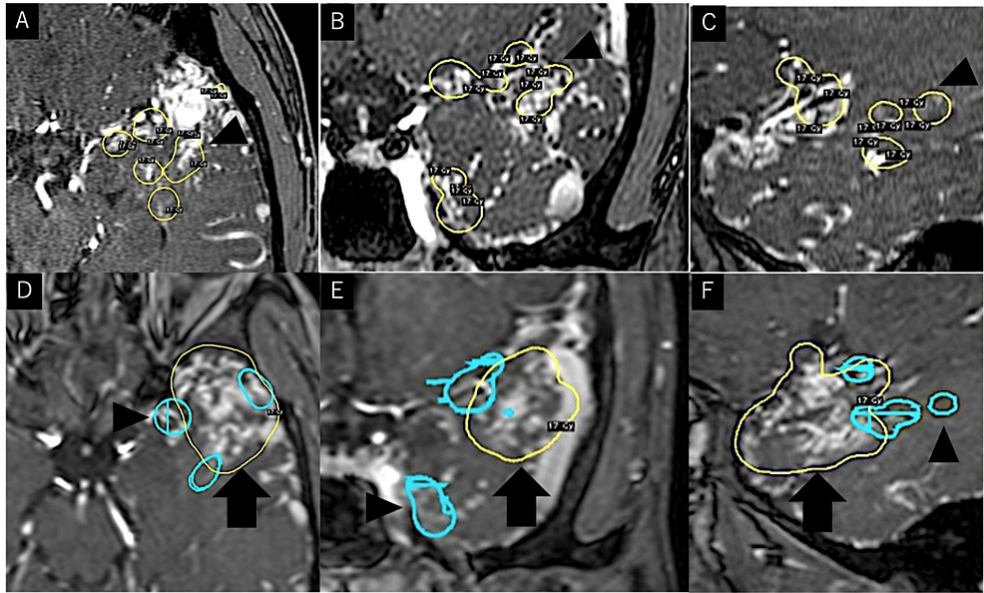
Snapshot of the first target in blue circles (black arrowheads) and the second target in yellow circles (black arrows) of Gamma Knife. (A-C) Axial, coronal, and sagittal images and 3D reconstruction showing the dose plan of the first GKS. The prescribed dose was 17 Gy (yellow line). (D-F) Axial, coronal, and sagittal images and 3D reconstruction showing the dose plan of the second GKS. The prescribed dose was 17 Gy (yellow line). Blue line shows the target of the first GKS. 3D, three-dimensional; GKS, Gamma Knife radiosurgery

The lesion was classified as a Spetzler-Martin grade Ⅲ AVM [7]. First, embolization of the feeder aneurysm of the AchA and flow reduction in the AVM via embolization of the left MMA, which is considered a component of the original AVM, were performed. Both procedures were performed using 30% N-Butyl-2-cyanoacrylate (Histoacryl®, Braun, Aesculap AG, Tuttlingen, Germany). The first session of the volume-staged Gamma Knife radiosurgery (GKS) was started two weeks after the embolization. The prescribed dose was 17 Gy. The GKS targets were feeder vessels that were identified using preoperative radiological imaging. Specifically, the estimated inflow areas were seven targets, and the target volumes of each were 0.47, 0.23, 0.07, 0.42, 0.18, 0.12, and 0.10 cm^3^, respectively. A second stage of the GKS with the same prescribed dose was performed three months later, which targeted the non-irradiated areas and, finally, the entire nidus. The target volume was 9.7 cm^3^ (Figure 4).

**Figure 5:**
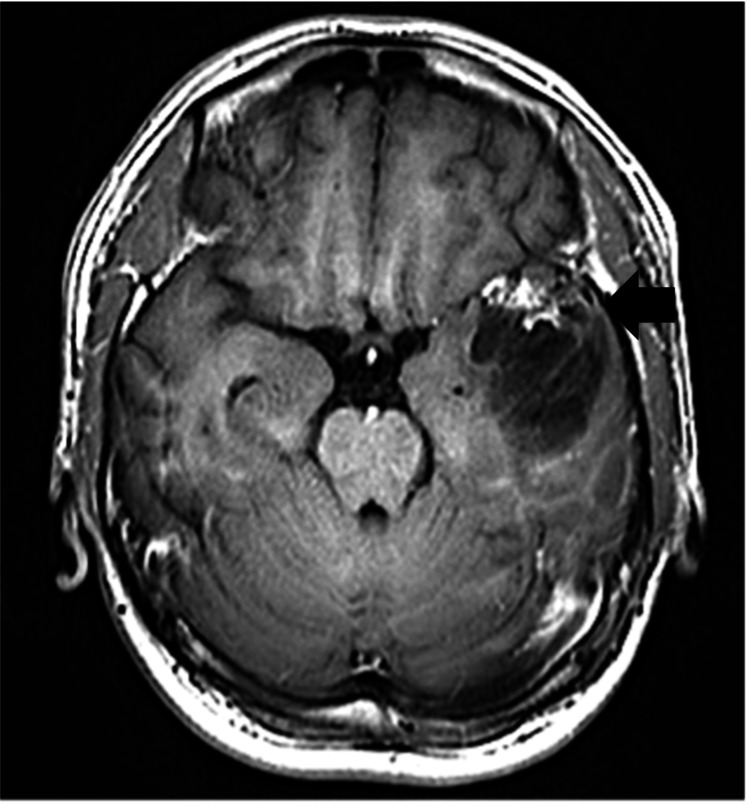
Axial magnetic resonance image showing a Gd-DTPA-enhanced lesion (black arrow) with perifocal edema appeared in the tip of the left temporal lobe. Gd-DTPA, gadolinium-diethylenetriaminepentaacetic acid

Three years after GKS was performed, a cyst was found near the nidus. The patient was administered antiepileptic medication five months after the cyst was detected because of a non-syncopal attack. When we examined the non-syncopal attacks, a gadolinium-diethylenetriaminepentaacetic acid (Gd-DTPA)-enhanced lesion with perifocal edema appeared at the tip of the left temporal lobe (Figure 5).

**Figure 6:**
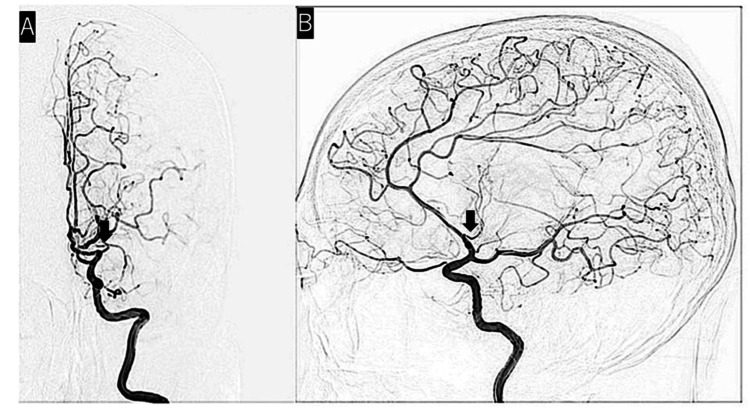
DSA image 4.5 years after GKS demonstrated no abnormal blood vessels other than the left M1 occlusion (black arrows). (A) Anteroposterior view of DSA. (B) Lateral view of DSA. DSA, digital subtraction angiography

Cerebral angiography performed 4.5 years after GKS showed no abnormal blood vessels other than the left MCA occlusion. It also showed complete occlusion of blood flow from MMA to AVM (Figure 6).

**Figure 7:**
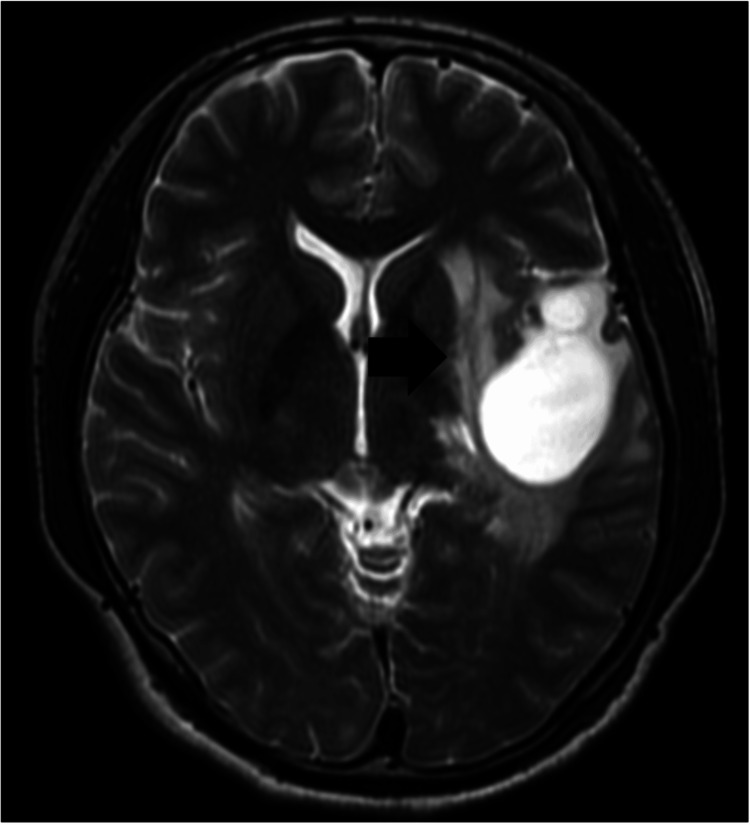
Axial magnetic resonance image of T2-weighted image showing perifocal edema (black arrow) getting bigger than 22 months ago, when the cyst was diagnosed.

Twenty-two months after the cyst was diagnosed, a lesion developed with edema (Figure 7).

Progressive cyst formation accompanied by a contrast-enhanced nodule was interpreted as a late radiation effect of GKS for AVM [8]. Surgery was conducted to remove the contrast-enhanced nodule and open the cyst as the lesion grew in size and progressed. The postoperative course was uneventful, and the patient had no complications one year postoperatively.

## Discussion

Mawad et al. [9] hypothesized that the high blood flow through the vessels in AVMs leads to intimal proliferation and stenosis. The exact frequency of this condition is unknown; however, AVM with major artery occlusion that is not unilaterally moyamoya, similar to the present case, is extremely rare, with only 15 cases, including the present case, being reported till date [1-6]. Enam and Malik [4] reported that seven (1.4%) of the 500 treated AVMs had similar pathologies. The internal carotid artery (ICA) is the most commonly occluded artery; however, the anterior carotid artery is also involved. Fourteen of these patients with marked arterial proliferation had the formation of moyamoya vessels ipsilateral to the AVM as a secondary change to arterial occlusion, similar to the present case.

Symptoms in AVM with major artery occlusion, including our case, were transient ischemic attacks (TIAs) in four cases, seizure in four cases, intracranial hemorrhage (ICH)+IVH in two cases, IVH in two cases, and ICH, subarachnoid hemorrhage, ischemia, stroke (details unknown), progressively declining memory and cognitive function, and proptosis in one case, each [1-6]. Ajiboye et al. [10] stated in their review article that ICH, seizures, and focal neurological deficits such as ischemia occur in 38-71%, 18- 40%, and 1-40% of AVMs, respectively. Table 1 shows that hemorrhagic events, seizures, and ischemic events account for 40%, 27%, and 33% of AVMs with major artery occlusion, respectively. Despite a small number of cases, these findings suggest that AVM with main artery obstruction may cause increased symptoms of ischemia. Surgical resection was reported in seven cases, radiosurgery in four cases, and STA (superficial temporal artery)-MCA bypass and resection in one case [1-6] (Table 1).

**Table 1:** Reported cases of AVM with major artery occlusion ACA, anterior cerebral artery; AchA, anterior choroidal artery; Acom, anterior communicating artery; AVM, arteriovenous malformation; cAVM, cerebral arteriovenous malformation; ECA, external carotid artery; F, female; GKS, Gamma Knife surgery; ICA, internal cerebral artery; ICH, intra cerebral hemorrhage; IVH, intra ventricular hemorrhage; L, left; LVO, large vessel occlusion; M, male; MCA, middle cerebral artery; MMA, middle meningeal artery; NA, not available; PCA, posterior cerebral artery; R, right; SAH, subarachnoid hemorrhage; SCA, superior cerebellar artery; SM grade, Spetzler-Martin grade; TIAs, transient ischemic attacks; VA, vertebral artery

Author/year	Age/sex	Presenting symptoms	Arterial occlusion	AVM location	Arterial supply of AVM	Treatment	Patient outcome
Solomon and Michelsen (1984) [1]	58/F	TIAs	L ICA at its supraclinoid portion	L subfrontal region + L parietal lobe	Acom and PCA	Resection of AVM	Hemorrhage at contralateral basal ganglia and planning to remove another AVM
Aoki and Mizutani (1985) [2]	38/M	ICH and IVH	R MCA at its origin	L parietotemporal region	Posterior temporal artery through a collateral supply from the ACA	Resection of AVM	Reoperation for postoperative hemorrhage in the brain surrounding the cAVM
Montanera et al. (1990) [3]	54/F	TIAs	R supraclinoid ICA	Bilateral frontal lobe	External carotid artery	Observation	NA
	44/M	TIAs	Occluded proximal R MCA	R parietal lobe	Perforating lenticulostriate arteries	Observation	NA
Enam and Malik (1999) [4]	37/M	Seizures, ischemia	R ICA occlusion	R frontal lobe	Collaterals from R ICA and posterior circulation	Resection of AVM	Improving of hemoparesis
	49/M	Seizures, stroke (details unknown)	R ICA occlusion	R frontal lobe	R MCA and R ACA through collaterals and ECA	Resection of AVM	No deficits
	67/F	ICH	R ICA	R Sylvian fissure	R PCA and R ACA	Resection of AVM	Death from coagulopathy secondary to excessive blood loss
	61/F	ICH and IVH	L ICA	L basal ganglia and thalamus	Anterior and posterior thalamoperforating arteries, lenticulostriate arteries	Radiosurgery	Neurological improvement
	39/M	Proptosis	R MCA	R temporal lobe	Through collaterals only. Dural component via bilateral ECA	Observation	Unchanged
	27/F	TIAs	L MCA (M1)	L parietotemporal lobe	Temporal branch of L PCA, posterior temporal and angular branches of L MCA	Resection of AVM	Uneventful
	66/M	IVH	R VA	Superior vermis	SCA and R PCA (the side of SCA is unknown）	Observation	Unchanged
Numaguchi et al. (2000) [5]	26/F	Seizure	L MCA (M1)	R basal ganglia and deep frontoparietal region	Pial leptomeningeal collateral circulation and moyamoya vessels	GKS	Uneventful, planned second GKS for residual nidus
	42/M	Seizures, progressively declining memory and cognitive function	L major trunk of the MCA	L temporal lobe	Moyamoya vessels, collateral pial branches of the ACAs and PCAs, and ECA branches.	Planned staged GKS	NA
Fassett et al. (2004) [6]	44/M	SAH	L MCA (M1) with distal reconstitution of the MCA branches through pial collateral vessels from the L ACA	L parietal lobe	L ACA and MCA	Resection of AVM	Uneventful
Present case	29/M	IVH	L MCA	L temporal	Branches of the L ACA, PCA, MCA, AchA, and MMA	GKS	Complete obliteration but needed craniectomy for cyst formation

Mawad et al. [9] suggested that surgical resection or embolization was not possible or was contraindicated in these cases because of the risk of worsening cerebral ischemia. Successful surgical resections have been described in several reports [1,2,4]. Solomon and Michelsen [1] reported a case of a left subfrontal AVM wherein the left ICA was occluded and the AVM was supplied by branches of the right ICA and the left external carotid artery. Postoperatively, the patient experienced a hemorrhage in the right basal ganglia. They speculated that this hemorrhage occurred due to the redirection of blood flow to the chronically ischemic brain after AVM resection. Aoki and Mizutani [2] described a case of posterior temporal AVM with right MCA obstruction. Despite successful AVM resection, massive cerebral edema can unexpectedly occur intraoperatively. Enam and Malik [4] removed three of their four AVMs without complications; however, one patient died of uncontrolled bleeding from a moyamoya vessel. Hemorrhagic complications or cerebral edema during or after surgical resection of this type of AVM are likely due to the normal perfusion pressure breakthrough phenomenon associated with surgical procedures for large, high-flow AVMs; therefore, these possibly occur after resection or embolization [11].

Enam and Malik [4] performed radiosurgery in one of seven patients, who showed progressive clinical improvement. Radiosurgery has been used as an effective treatment for AVMs, with our case having a mean nidus diameter of 26.5 mm; approximately 70-80% of AVMs less than 3 cm disappear during radiosurgery [12]. Although GKS in our case resulted in the disappearance of the AVM and of moyamoya-like vessels without ischemic attacks, cyst formation was observed postoperatively. Shuto et al. [8] reported 18 (2.3%) cases associated with delayed cyst formation of the 775 patients with AVM who underwent GKS. No correlation with age, sex, nidus volume, margin volume, preembolism followed by GKS, or hemorrhage before GKS was noted. Nine patients required surgical treatment, eight underwent craniotomy to remove vascular lesions, and one underwent cyst aspiration. There were no lesion recurrences in the eight patients who underwent craniotomy. Cysts that develop after GKS in AVM are enlarged due to repeated mild hemorrhages because of nodular vascular lesions that develop primarily within adjacent brain regions, and the optimal treatment is craniotomy to remove the vascular lesions by releasing the cyst, as we did in our case [13].

AVMs with major arterial obstruction and consequently decreased vascular reserves are difficult to resect and may lead to serious complications. There are insufficient data to determine the optimal treatment; however, radiosurgery might be a safe treatment approach for AVMs under these conditions. There is a possibility that steady progressive changes in the vasculature after radiosurgery can gradually reduce misery perfusion, decreasing the risk of interrupting collateral vessels between the internal and external carotid artery systems, which increases AVM [14].

## Conclusions

We encountered a case of an AVM with ipsilateral major feeding artery occlusion that was treated with combined embolization and GKS without irreversible sequelae. AVM with major artery occlusion that is not unilateral moyamoya disease, similar to the present case, is extremely rare, with only 15 cases reported, including the present case. GKS appears to be safe and effective for AVM with ipsilateral major vessel occlusion.
